# Evolution of self-organised division of labour driven by stigmergy in leaf-cutter ants

**DOI:** 10.1038/s41598-022-26324-6

**Published:** 2022-12-20

**Authors:** Viviana Di Pietro, Patrick Govoni, Kin Ho Chan, Ricardo Caliari Oliveira, Tom Wenseleers, Pieter van den Berg

**Affiliations:** 1 Laboratory of Socioecology and Social Evolution, Department of Biology, KU Leuven, Naamsestraat 59, 3000 Leuven, Belgium; 2 Dynamics in Biological Systems Lab, Department of Cellular and Molecular Medicine, KU Leuven, Herestraat 49, 3000 Leuven, Belgium; 3 Laboratory of Biodiversity and Evolutionary Genomics, Charles Deberiostraat 32, 3000 Leuven, Belgium; 4 Departament de Biologia Animal, de Biologia Vegetal I d’Ecologia - Universitat Autònoma de Barcelona, 08193 Bellaterra, Barcelona Spain; 5 Evolutionary Modelling Group, KU Leuven, Naamsestraat 59, 3000 Leuven, Belgium

**Keywords:** Evolution, Behavioural ecology, Ecological modelling

## Abstract

Social insects owe their widespread success to their ability to efficiently coordinate behaviour to carry out complex tasks. Several leaf-cutter ant species employ an advanced type of division of labour known as task partitioning, where the task of retrieving leaves is distributed between workers that cut and drop and those that collect the fallen leaves. It is not entirely clear how such highly coordinated behaviour can evolve, as it would seem to require the simultaneous mutations of multiple traits during the same generation. Here, we use an agent-based simulation model to show how task partitioning in leaf-cutter ants can gradually evolve by exploiting stigmergy (indirect coordination through the environment) through gravity (leaves falling from the treetop on the ground forming a cache). Our simple model allows independent variation in two core behavioural dimensions: the tendency to drop leaves and the tendency to pick up dropped leaves. Task partitioning readily evolves even under these minimal assumptions through adaptation to an arboreal environment where traveling up and down the tree is costly. Additionally, we analyse ant movement dynamics to demonstrate how the ants achieve efficient task allocation through task switching and negative feedback control.

## Introduction

Insect societies of ants, bees, wasps and termites are widespread and dominate nearly every environment on earth^1^. One of the reasons for their remarkable ecological success is their ability to perform tasks in a coordinated fashion^2^. While the behaviour of a single individual is simple in nature, dynamic interactions among individuals and between individuals and their environment can lead to the emergence of a variety of complex and adaptive collective behaviours^3–8^.

The functioning of a social insect colony requires performance of a wide range of tasks. While some tasks can be performed by single individuals (*e.g.*, foraging in most bees and wasps^2,9,10^), others require many individuals to work in parallel (*e.g.*, building a nest^2,11^). Some highly eusocial species exhibit task partitioning, where sequential actions are carried out by different sets of individuals, which requires an accurate temporal division of labour^11–14^. Task partitioning reduces delays caused by individuals switching tasks, allowing for increased work efficiency^15,16^.

One of the factors that can modulate coordination between individuals is an indirect communication process called stigmergy^17,18^. In this process, individuals leave traces or modify the environment in a way that alters the behaviour others that perceive these changes. The environment, therefore, documents and organizes collective behaviour, driving coordination without the need for direct communication^17^. *Polistes* wasps build their nest according to a stigmergic mechanism where individuals tend to build new hexagonal cells in between existing nest cells^19,20^. Over time, this mechanism ensures the emergence of a coherent nest architecture, even if the building activity is conducted concurrently by different individuals^19,20^. Stigmergy is also the central principle behind ant trail building, where pheromones dropped by one ant indirectly attract surrounding ants, stimulating further pheromone deposition in a behavioural positive feedback loop. The self-organized, collective behaviour or pattern that emerges is what we know as an ant trail^21^. Other examples of such decentralized, collective behaviours that emerge through stigmergy include brood sorting (in bees^22,23^ and ants^24–26^), corpse disposal (in ants^27,28^), foraging patterns (in bees,^29^ and ants^30–32^) and mound building in termites^33^.

**Figure 1:**
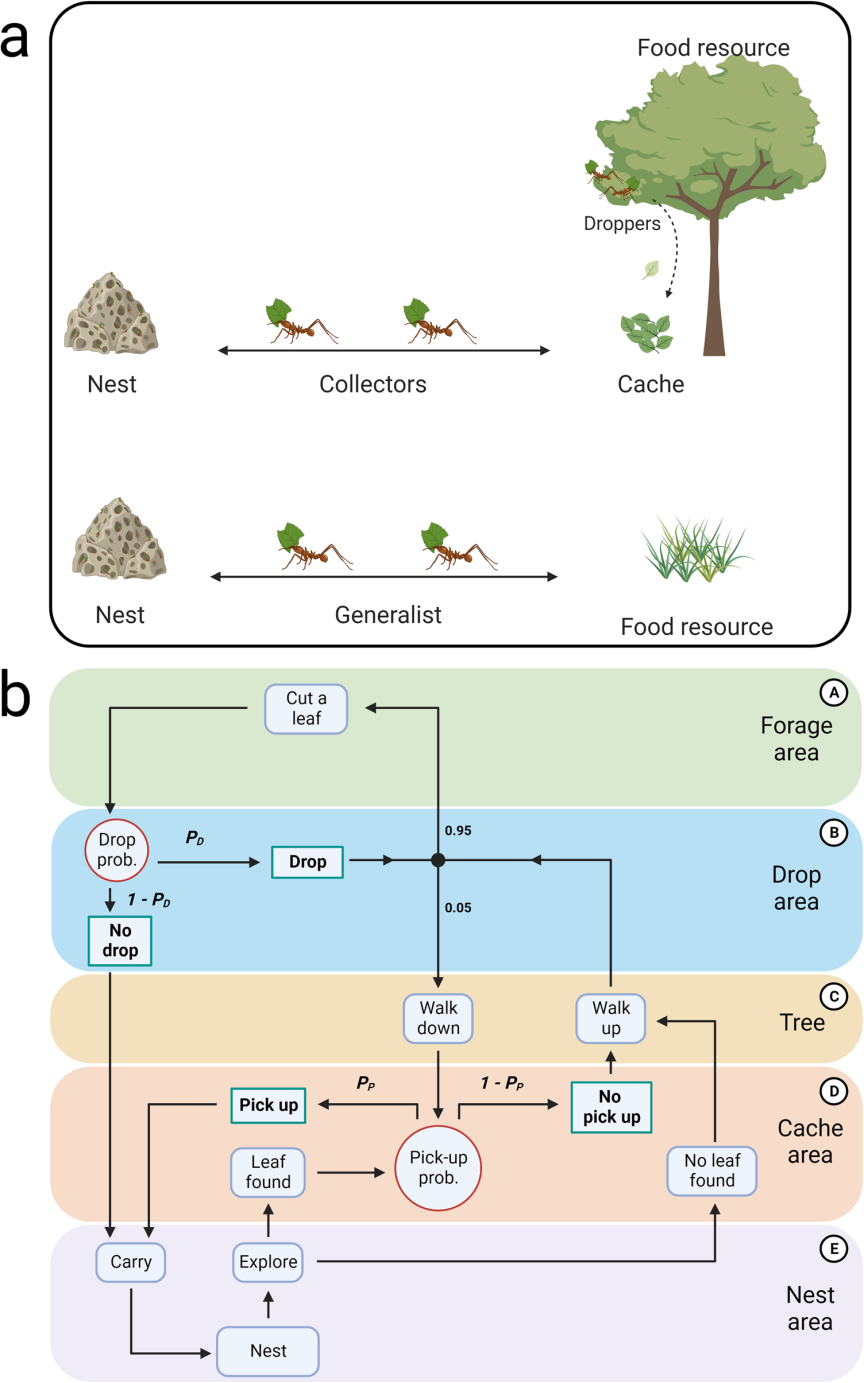
Overview of the model system and simulation design. (**a**) Visual representation of the two types of foraging strategies observed in leaf-cutter ants, task-partitioned (top) and generalist (bottom). Under task partitioning, individuals either cut and drop leaves from the treetop (‘droppers’) or collect and return the dropped leaves on the ground (‘collectors’). In contrast, generalists individually cut and return leaves to the nest. (**b**) Schematic representation of the model showing the different areas of the arena and the corresponding ant decisions and actions in each area. The red circles indicate the two key behavioural variables in the model: the tendency to drop leaves from the tree (given by the ‘drop probability’, *P*_*D*_) and the tendency to pick up leaves in the cache area (given by the ‘pick-up probability’, *P*_*P*_). Depending on these probabilities, the ant will decide to act or not, indicated by the green boxes. A third decision node in the *Drop area* (B) controls forage probability, fixed at 0.95, to implement a small amount of noise in the system, allowing the ants to dynamically balance flows between the areas. Created with BioRender.com.

A particularly interesting behavioural difference can be observed between leaf-cutter ant species (*Atta* and *Acromyrmex* spp.) that are ground-foraging as opposed to those that retrieve leaves from arboreal environments. While ground-foraging species typically cut and retrieve grass or seeds as an individual task, those that collect leaves from trees split the task of leaf retrieval, with one set of ants cutting and dropping leaf fragments from the tree and other workers then collecting the leaf fragments as they accumulate on the ground in a leaf cache^34,35^ (Fig. 1a). Dropped leaves act as a stigmergic stimulus for the foraging ants, inducing them to collect from the cache whenever leaves are found. When foraging on trees, task partitioning harnesses gravity to reduce the energy and time costs associated with climbing up and down the tree, while a generalist strategy would be expected to be most efficient for ground-foraging species. However, task partitioning efficiency can be affected by accumulated transfer-related costs (i.e. time and effort of finding the dropped leaves or loss of leaves that are dropped but not found) or by trading off a high leaf delivery rate for enhanced information transfer on food quality (e.g. sequential load transport in *Atta vollenweideri*^36^, *Acromyrmex crassispinus*^37^ and *Acromyrmex subterraneus subterraneus*^37^).

Whereas the evolutionary advantages of adopting task partitioning are evident, particularly in the case of arboreal leaf-cutter ants foraging on sufficiently tall trees, it remains unclear how this phenomenon could have evolved. At first sight, it seems like the evolution of task partitioning would require several complex traits to evolve simultaneously, including the capacity to split the tasks into subtasks and the capacity to execute these subtasks in a coordinated fashion. Although a previous model has shown that task allocation can evolve without any pre-adapted building blocks^38^, the open behavioural architecture of this model (that made use of a grammatical evolution approach) and high mutation rate used might have allowed the simultaneous origin of several traits during the same generation, even if this would typically not be plausible in real biological systems. In addition, the relation between task partitioning and the involved core actions (dropping and picking up of leaves), how these two actions carried out by separate sets of individuals may coevolve, as well as how these actions may dynamically coordinate collective behaviour, remains unclear. This last point is particularly pertinent in social insect behaviour, where the lack of a central decision-making structure necessitates the integration of individual interactions to effectively respond to a variable environment^5–7,39^.

In this study, we take a minimalist approach, only allowing the independent evolution of two core behaviours: the probability to drop a leaf and the probability to pick up a dropped leaf. In this way, we study how task partitioning can gradually evolve through the simultaneous evolution of independent mutations, without requiring biologically implausible mechanisms such as high mutation rates. We study the coevolution of these two behaviours with respect to the potential for efficiency gain (given by the height of the tree), hypothesizing that task partitioning more readily evolves in arboreal environments with taller trees. Finally, we analyse patterns in ant movement dynamics that emerge in each investigated scenario to gain a more mechanistic understanding of how the ants collectively achieve efficient foraging strategies.

## The model

We developed a simple agent-based model^40^ inspired by the foraging behaviour observed in leaf-cutter ants. We designed a 2D arena (shown in Supplementary Fig. S1) consisting of a grid of cells in which 25 foraging ants could move around, collect leaves from a tree, and bring them to the nest over a period of 10,000 timesteps. The arena was partitioned (as illustrated in Fig. 1b) into the *Forage area* (A) on the top of the tree, where ants could collect leaves, the *Drop area* (B) where ants could decide to drop collected leaves, the *Tree area* (C) which represents the trunk of the tree (the height of which could vary, see below), the *Cache area* (D) where dropped leaves accumulated and ants could pick them up, and the *Nest area* (E) where ants could deliver collected leaves. The total number of leaves brought to the nest by the end of the simulation was used as a proxy for colony fitness.

Initialized in random locations around the grid, the ants move one cell per simulation time step in a direction decided by whether they are carrying a leaf, whether they detect a leaf (within 5 cells from their current position), and according to their recent history. If the ants detect a leaf in the *Cache area*, they walk towards it and pick it up according to their pick-up probability (*P*_*P*_), walking towards the nest as soon as they picked up the leaf. If they do not detect a leaf or decided to not pick it up, they walk towards the *Forage area.* Here, they either cut and carry a leaf (with 95% probability) or climb down the tree toward the nest without carrying a leaf (with 5% probability; a behaviour observed in *Atta columbica*^41^ that allows flexibility in the ratio of droppers to collectors over time). Upon moving into the *Drop area*, they drop the leaf (which would then appear in the *Cache area*) according to the drop probability (*P*_*D*_).

We systematically and independently varied *P*_*D*_ and *P*_*P*_ to obtain an overview of the colony fitness associated with these variables, depending on the tree height. The colony paid a fitness cost linearly associated with *P*_*P*_, reflecting the assumption that ants spend time and energy looking for food items at the at the *Cache area* (see Methods for details). On the other hand, *P*_*D*_ has a cost intrinsically embedded in the model: dropping leaves that are not picked up results in a steadily increasing unused cache, wasted time and energy, and a severely affected colony fitness. This cost is parallel to that associated with leaves that are dropped but not found due to wind, rain, or other complicating factors. We ran all simulations for three different environments: a terrestrial, ground-foraging environment (a tree height of 1 cell), an intermediate environment (three height 10), and an arboreal environment (tree height 20). Having obtained fitness landscapes with respect to both behavioural variables for each tree height, we plotted the geographic distribution of the ants over time for the generalist and task partitioned strategies and ran evolutionary simulations to verify if the fitness maxima in the landscapes can be attained (see Methods for details).

## Results

**Figure 2:**
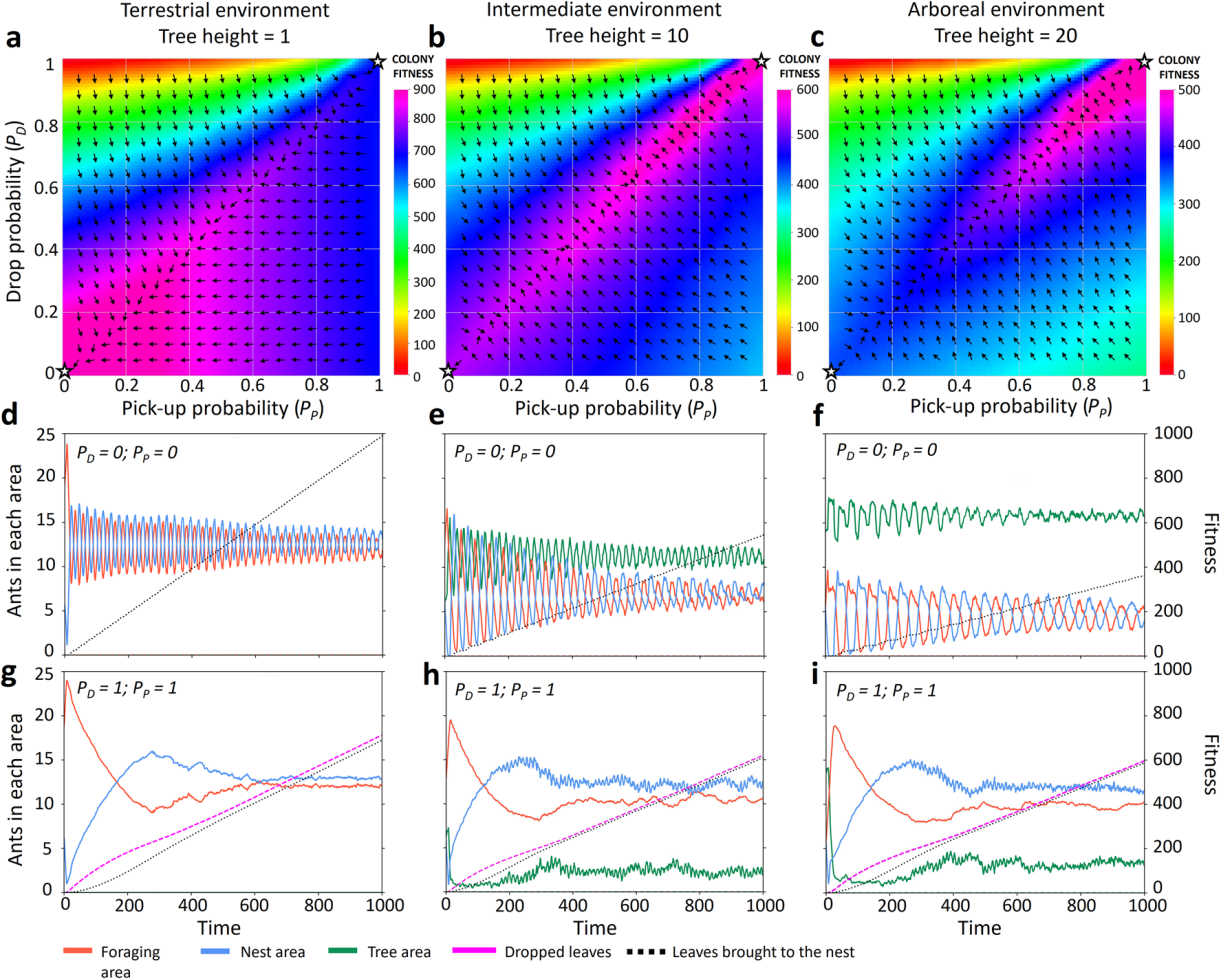
Fitness landscapes and collective movement plots. The topmost graphs show the fitness of colonies foraging in terrestrial (**a**), intermediate (**b**) and arboreal (**c**) environments, depending on pick-up probability (*P*_*P*_) and drop probability (*P*_*D*_). Colours indicate colony fitness, while the arrows indicate the fitness gradient. Ten replicate simulations were run, averaged, and linearly interpolated for all possible combinations of *P*_*P*_ and *P*_*D*_ from 0 to 1 with intervals of 0.05 for each environment. The middle graphs (**d**,**e**,**f**) show the locations of the ants over the first 1,000 time steps of 50 replicate simulations in each environment for a colony using the generalist strategy (*P*_*P*_ = *P*_*D*_ = 0; corresponding to the star in the bottom left corner of the upper graphs), while the bottom graphs (**g**,**h**,**i**) show the same for a colony using a fully task-partitioned strategy (*P*_*P*_ = *P*_*D*_ = 1; star in the top right corner). In these graphs, ants in the *Cache area* and the *Drop area* were respectively included in the counts for the *Nest area* and *Forage area*.

The fitness landscapes in Fig. 2 show the impact of tree height on the relative effectiveness of generalist and task partitioned foraging strategies. In a terrestrial environment, a fully generalist strategy that never drops leaves or picks up dropped leaves has the highest fitness, as shown in the lower-left corner (Fig. 2a). In an intermediate environment, fitness changes only slightly along the diagonal (where drop and pick-up probabilities are equal), indicating the two strategies have similar foraging productivities (Fig. 2b). Finally, in an arboreal environment, the highest fitness is observed on the upper-right corner, suggesting that task partitioning is favoured (Fig. 2c). In this scenario, fitness gradually increases from the bottom left (generalist) to the top right (task-partitioned) corner, suggesting that task partitioning can gradually evolve from a fully generalist strategy in our model (see also Supplementary Fig. S2 for the locations through time of single ants under both a generalist and a task-partitioning strategy).

Movement and behavioural dynamics among the various zones of the grid are illustrated by the collective activity graphs (Fig. 2d-i). Increasing tree height strongly affects the productivity of the generalist strategy (*P*_*P*_ = *P*_*D*_ = 0; middle panels). As the ants spend more time walking up and down the tree, less time is spent foraging and returning leaves to the nest. This is reflected by the fact that an increasing proportion of the ants is on the tree at any given moment (green lines). As a consequence, increasing tree height leads to reduced fitness of the generalist strategy (compare the bottom left corners in Figs. 2a-c). A notable characteristic of generalist movement dynamics is the oscillation, or exchange, between ants in the *Forage area* and *Nest area*. The amplitude of the oscillations, although initially high, dampens due to movement stochasticity averaging out over time. The populations in the two key areas oscillate around the same number of ants, regardless of the environment, demonstrating how it takes equal time to forage as it does to return leaves to the nest.

In contrast, the task-partitioned strategy is robust to increasing tree height and characterized by more stable collective dynamics (*P*_*P*_ = *P*_*D*_ = 1; bottom panels). After an initial spike of dropper ants in the *Forage area* results in an accumulation of dropped leaves in the *Cache area*, collector ants in the *Nest area* rebound before settling into an equilibrium with the droppers, when the flow of ants walking up matches the flow walking down, balancing coordination between the two tasks. The slightly greater steady state population in the *Nest area* is explained by the inefficiency of multiple ants finding and walking to the same leaf, which is not an issue in the *Forage area* due to relative abundance. Significantly, while the fitness of the generalist strategy decreases with increasing tree height, task-partitioned fitness is robust, allowing the ants to adaptively coordinate when foraging in an arboreal environment.

**Figure 3:**
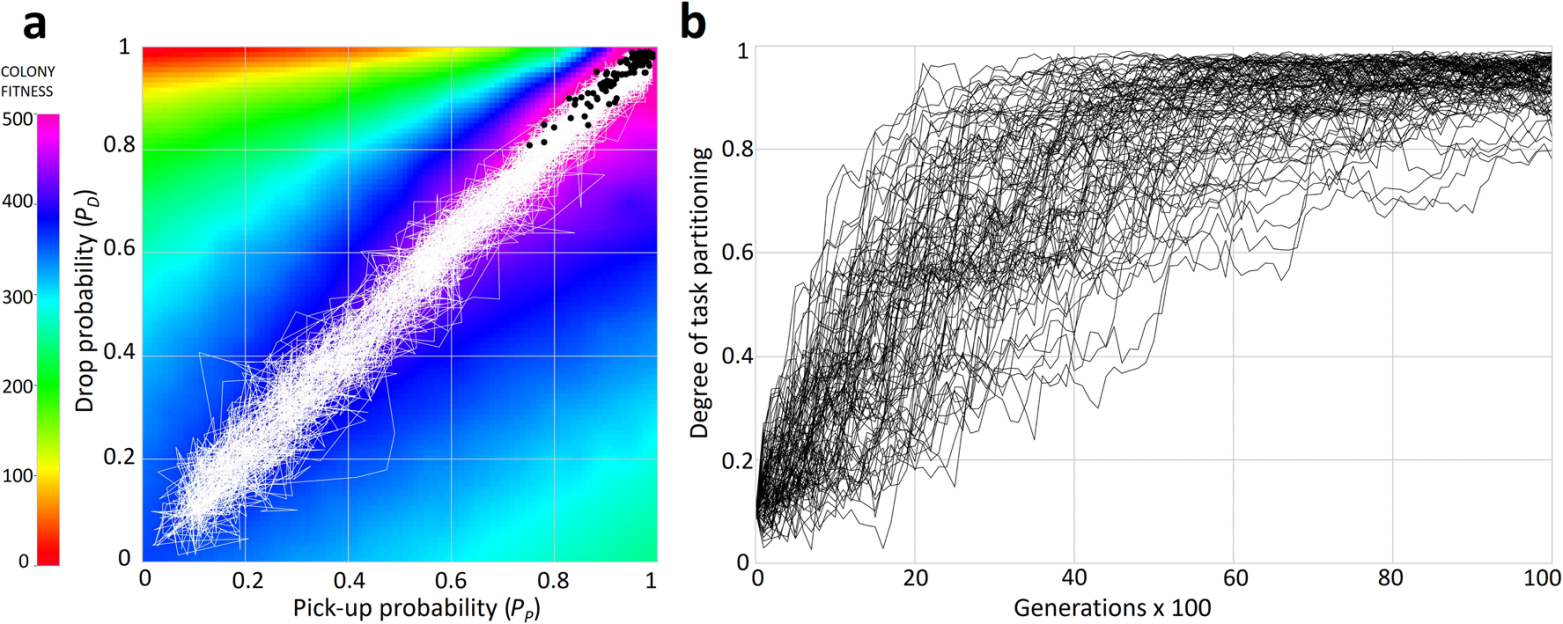
Evolutionary simulation. (**a**) Evolutionary trajectories (white lines) of 100 replicate simulations of 50 colony populations initialized with *P*_*P*_ and *P*_*D*_ between 0 and 0.2, plotted on the arboreal fitness landscape. Black dots represent evolved outcomes after 10,000 generations. (**b**) Degree of task partitioning, defined as the average between *P*_*P*_ and *P*_*D*_, plotted as a function of evolutionary time for the same 100 simulations.

Evolutionary simulations confirm that a task-partitioned strategy can gradually evolve from a fully generalist strategy in an arboreal environment. (Fig. 3; see Supplementary Fig. S3, S4 for the other tree heights). For an intermediate environment, the selection gradient is approximately flat, resulting in a wide distribution of strategies along the diagonal where *P*_*P*_ = *P*_*D*_ (Supplementary Fig. S4). As expected, in the terrestrial, ground-foraging environment, colonies remain concentrated in the lower-left corner, confirming a generalist strategy is in that case most rewarding (Supplementary Fig. S3).

## Discussion

In this paper, we developed a model simulating a foraging scenario in leaf-cutter ants to gain insight on the evolutionary basis of self-organized division of labour. Our model shows how task partitioning can evolve gradually from a minimal set of traits, even if colonies are genetically, morphologically and behaviourally homogeneous. We observed that the ants reliably evolve towards a task-partitioned strategy when there are efficiency gains to be made through stigmergic coordination, i.e. in an arboreal environment. This strategy is characterized by an equilibrium that emerges from environmental and behavioural feedback, in which some individuals end up predominantly cutting and dropping leaves at the crown of the tree, whereas others pick up the dropped leaves at the bottom of the tree and bring them back to the nest.

Our simulations show that a fully generalist strategy can gradually evolve towards a task-partitioned strategy without the need for the simultaneous emergence of multiple adaptive mutations or pleiotropy (where mutations of a single locus affect more than one trait simultaneously). Even if the probabilities to pick up and drop leaves mutate independently and in small steps, evolution reliably produces task-partitioning in environments where there is scope for efficiency gain. This reflects recent empirical research that finds ant collective behaviour to indeed be shaped by adaptive evolution^42–44^.

Previous models, not only involving social insects, showed that task allocation increases individual efficiency and reduces costs associated with switching tasks^38,45–49^. In line with these studies, our model shows that a dynamically coordinated form of task allocation readily evolves when switching costs are sufficiently high (in our case, in arboreal environments), while a generalist strategy is favoured when they are low (in our case, in terrestrial environments). In reality, tree height is not the only determinant of the adaptiveness of task-partitioning strategy. Task partitioning with direct transfer or cache formation has also been observed in ground-foraging species, when food resources are far from the nest location^50^. In such cases, cache formation can be due to high trail traffic, intersections between human and ant trails or changes in forager speed^35^. Overall, our findings add to previous evidence of the efficiency advantages of this type of division of labour^51^ and show that it can gradually evolve from a non-partitioned state^38,52^.

Several previous studies have made use of embodied multiagent simulations with relatively open behavioural architectures, for example based on artificial neural networks or grammatical evolution, to gain insight into the evolution of social behavioural strategies such as cooperation and communication across a wide range of domains, from ecology and neuroscience to robotics and engineering^38,53–56^. This has the clear advantage that the evolutionary process is relatively unconstrained by specific assumptions, and therefore in principle able to evolve a large range of behavioural reaction norms^57^. At the same time, such architectures may be subject to ‘soft constraints’^58^ (making some phenotypes more likely to evolve than others) that are often not obvious from considering the architecture alone, and therefore can suffer from some hidden biases and assumptions that one would not encounter with simpler architectures. Furthermore, understanding the drivers that underlie evolution in such open behavioural architectures is often far from straightforward. Our study should be considered complementary to previous models of division of labour with such open architectures^38^. While our genotype space is highly constrained (two-dimensional), it does clearly show how two core behavioural dimensions (picking up and dropping leaves) can coevolve to produce self-organized division of labour.

In our model, colonies are genetically homogeneous, meaning that all individuals within a colony have identical pick-up and drop probabilities and have identical morphologies. Whereas in reality, both genetic and morphological heterogeneity among foragers is present in leaf-cutter ant species^59–63^. While in some species, ants with differing phenotypes in some species engage in entirely distinct tasks, such as brood care and foraging, other species produce a range of forager ant sizes that specialize in different vegetation. In the case of *Atta cephalotes*, heterogeneity in foragers size emerges only at large colony sizes, suggesting that the benefits of specialization, and potentially individual-stable task allocation, might increase with increasing access to resources^59^. This may be related to the effect of mismatches between load size and carrying capability in sequential load transport, which has been shown to negatively affect leaf transportation rate^36,37^. Also, as colonies grow, they tend to shift from foraging on small herbs to foraging on tall trees^59^. This might be because efficient foraging on tall trees via task partitioning requires a suitably large colony to sustain flow along the tree between dropper and collector roles^59,64^. In view of these considerations, interesting extensions to our model would include phenotypic specialization, the explicit modelling of multiple components that contribute to fitness, and allowing colony size to change over time.

Leaf-cutter ants belong to a group (Attini tribe) of over 200 fungi-growing ant species, most of which do not cut leaves but collect debris from the ground to feed the fungus^64^. The transition to leaf cutters (*Atta* and *Acromyrmex*, 8–12 million years ago) coincided with relative ecological dominance from forest to grassland and was followed by many changes in colony organization such as the emergence of polymorphic worker castes and an increase in colony size, as well as the evolution of advanced task allocation to process food resources^64,65^. This shift suggests terrestrial grass-foraging preceded that of arboreal leaf-foraging, aligning with our assumptions^64,66^, and that dropping and collecting behaviours coevolved as a result of climbing and the costs associated. Therefore, if ants follow a generalist strategy, they only focus on cutting leaves from the tree or grass themselves, not paying the cost of spending time looking for pre-processed fragments. On the other hand, when leaves are dropped, ants will invest more time looking for those leaves and coevolution of the two behaviours is needed to dynamically coordinate efficient division of labour.

Further empirical study of leaf-cutter ant foraging is needed to gain a more comprehensive understanding of how individual behavioural mechanisms give rise to collective patterns. We focused on stigmergy via leaves as the main method of communication, leaving out any kind of direct communication or recruitment signals to affect behaviour. However, signals such as pheromones or stridulation may significantly impact division of labour and movement^67,68^. In field quantification, foraging *Atta* ants were estimated to not pick up approximately half of the leaf fragments dropped^34,69^. It is unclear if this is due to limited detection ability, the decision to focus on competing tasks, or other environmental variables. However, although a percentage of leaves are lost, it has been shown that using task partitioning is rewarding if the ratio of the cost of the carriers to that of the droppers is smaller than the ratio of the number of leaves successfully retrieved to the number of lost leaves^69^. This would be the case when the cost of processing the leaf material at the bottom of the tree is compensated by saving multiple trips up and down on tall trees^69^, which means that our results should broadly hold for relatively tall trees.

The foraging dynamics exhibited by our model closely align with previous empirical studies and theoretical models of ant foraging and task allocation^70–77^. In these studies, complex behavioural dynamics emerge due to local interactions and feedback loops in order to uniformly distribute tasks within the colony. In our case, the cache of dropped leaves is controlled by task switching coupled with negative feedback between the cache and collector ants: more dropped leaves attract collectors, depleting the cache, until the collector ants are forced to abandon their task, walking up the tree to replenish the cache. These homeostatic mechanisms resemble an integral control scheme^75^ as put forth in a recent social insect model as well as several adaptive biochemical and neuronal networks^78–82^. As predicted in the above studies, delay due to the time taken to switch tasks (walking the tree) can cause oscillations in the feedback network (Supplementary Fig. S5). Moreover, the ants collectively move in similar patterns regardless of initialization assumptions (Supplementary Fig. S6). Given the simplicity, robustness, and prevalence of negative feedback control, it is possible that these dynamics are fundamental to the task of stigmergic foraging with a dynamic division of labour. Our stigmergic task switching dynamics parallel those modelled from chemical and tactile interactions, demonstrating the essential nature of negative feedback to coordinating task allocation^77^. Similar dynamics emerge by evolving neural network-controlled agents to balance two objectives in a dynamically changing environment, supporting the hypothesis^83^ that task switching via negative feedback is central to efficient division of labour.

Our results show how a seemingly complex group-level phenomenon such as task partitioning can arise from a minimal behavioural architecture via the coevolution of two independent behavioural variables. We have highlighted the potential importance of stigmergy, tree height and negative feedback control to drive the functioning of task partitioned foraging in leaf-cutter ants. Our work provides a foundation to help generate experimentally testable hypotheses for how this behavioural phenomenon may have evolved.

## Methods

### Environment and agents

The model was coded in Python v3.9 using the “*Mesa*” package^84^ developed for agent-based models. The size of the 2D grid depended on the environment considered: 11 × 16 cells for a terrestrial environment with tree height = 1, 11 × 25 cells for an intermediate environment with tree height = 10, and 11 × 35 cells for an arboreal environment with tree height = 20 (Supplementary Fig. S4: example grid for an arboreal environment). The vertical sizes of each area except the *Tree area* were kept constant between the different environmental scenarios: 7 cells for the *Forage area*, 1 for the *Drop area*, 1 for the *Cache area*, and 7 for the *Nest area*. The nest comprised a single cell and is located at (6, 3).

The forager ants are autonomous agents, initialized in random locations around the grid, moving one cell per time step. Movement with a leaf is calculated in straight lines down the tree to the nest, mimicking path integration, while without a leaf, the ant stochastically moves up or down the tree (by picking forward, diagonal, or horizontal cells in a uniform distribution) to simulate olfactory noise^85^.

### Colony fitness

The fitness of a colony was determined by the total number of leaves the ants collected over a total of 10,000 time steps. In addition, we assumed that colonies paid a fitness cost that was linearly associated with *P*_*P*_, as follows:

$$\omega ~ = ~W~{-}~c*~P_{P}$$

where *ω* denotes colony fitness, *W* denotes the total amount of leaf fragments brought back to the nest and *c* denotes the magnitude of the fitness costs associated with *P*_*P*_. For all simulations shown, *c* = 0.3.

### Simulation set-up

To construct the fitness landscapes, we ran 10 replicate simulations for all possible combinations of *P*_*P*_ and *P*_*D*_ from 0 to 1 with intervals of 0.05 for each environment (for a total of 441 combinations of *P*_*P*_ and *P*_*D*_ for each environment). We averaged the resulting fitness over the replicates per parameter combination, and performed a linear interpolation on these averages to create the fitness landscapes (top panels of Fig. 2a–c).

For the evolutionary simulations, we constructed populations of 50 colonies that were each initialized with values for *P*_*P*_ and *P*_*D*_ randomly drawn from a uniform distribution in the interval 0 and 0.2. Generations were non-overlapping and population size was held constant throughout the generations. Colonies each obtained a fitness (*ω*) based on their values of *P*_*P*_ and *P*_*D*_ that were extracted from the landscapes previously produced. Colonies were assumed to reproduce sexually, proportionally to their fitness. For simplicity, we assumed haploid genetics and that the two loci were unlinked. Offspring colonies randomly and independently inherited the values of *P*_*P*_ and *P*_*D*_ from one of their parent colonies, with a small chance of mutation (0.01) that was independent between the two loci. Mutations at both loci were implemented by drawing a number from a normal distribution with mean 0 and standard deviation 0.1 to the parent value, truncated to the range 0–1. All simulations were run for 10,000 generations. We ran 100 replicate simulations for each environment.

## Supplementary Information

Supplementary Information.

## Data Availability

The data and codes generated during the current study are available in the Mendeley Data repository, https://doi.org/10.17632/njms3jnbp4.2. The code to reproduce our model is also freely available via: https://pgovoni21.github.io/ants-task-partitioning-ABM/.
