# Supplementary material for: Evolution of self-organised division of labour driven by stigmergy in leaf-cutter ants
Source: Sci Rep. 2022 Dec 20;12:21971. doi: 10.1038/s41598-022-26324-6 (PMC9768137; doi:10.1038/s41598-022-26324-6)
Supplement: Supplementary file 1 — Supplementary Information. [file 41598_2022_26324_MOESM1_ESM.docx]

**Supplementary Information**

**Title**: Evolution of self-organised division of labour driven by stigmergy in leaf-cutter ants

**Authors**: Viviana Di Pietro^1†*^, Patrick Govoni^2†^, Kin Ho Chan^3^, Ricardo Caliari Oliveira^1,4^, Tom Wenseleers^1^, Pieter van den Berg^5^

^1^ Laboratory of Socioecology and Social Evolution, Department of Biology, KU Leuven, Naamsestraat 59, 3000 Leuven, Belgium

^2^ Dynamics in Biological Systems Lab, Department of Cellular and Molecular Medicine, KU Leuven, Herestraat 49, 3000 Leuven Belgium

^3^ Laboratory of Biodiversity and Evolutionary Genomics, Charles Deberiostraat 32, 3000 Leuven, Belgium

^4^ Departament de Biologia Animal, de Biologia Vegetal i d'Ecologia - Universitat Autònoma de Barcelona

08193 Bellaterra (Barcelona), Spain

^5^ Evolutionary Modelling Group, KU Leuven, Naamsestraat 59, 3000 Leuven, Belgium

^†^These authors contributed equally

^*^Corresponding author: Viviana Di Pietro (viviana.dipietro@kuleuven.be)


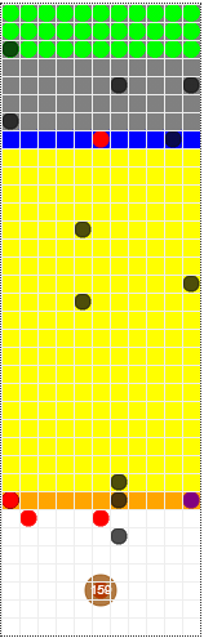


Supplementary Figure S1. **Overview of the 2D arena.** Leaves are represented by the green dots in the *Forage area* (in grey). Black dots are the ants moving around looking for leaves, and they become red once a leaf is collected. Ants can decide to drop a leaf when they cross the *Drop area* (in blue). Purple dots are leaves dropped in the *Cache area* (in orange). Between the *Drop area* and the *Cache area* there is the *Tree area* (in yellow), in this example with a tree height of 20. The nest is placed in the *Nest area* (in white) at location (6, 3).


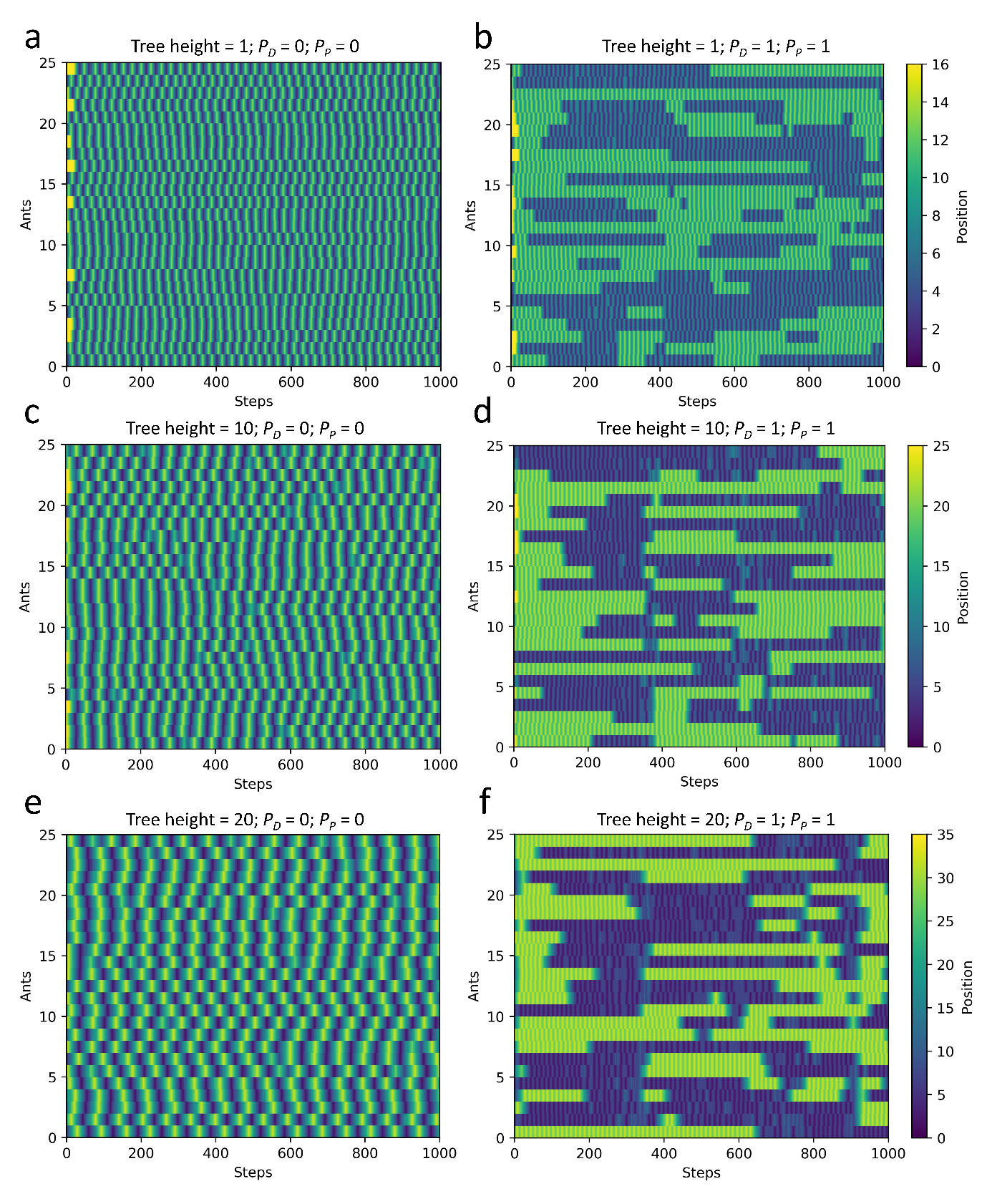


Supplementary Figure S2. **Individual movement dynamics.** The graphs show the geographical position of the ants in the arena for each environmental scenario, comparing generalist (a, c, e) and task partitioning (b, d, f) strategies. Colours indicate the different zones of the arena, in which blue shades indicate the *Nest area* while yellow shades indicate the *Forage area*.


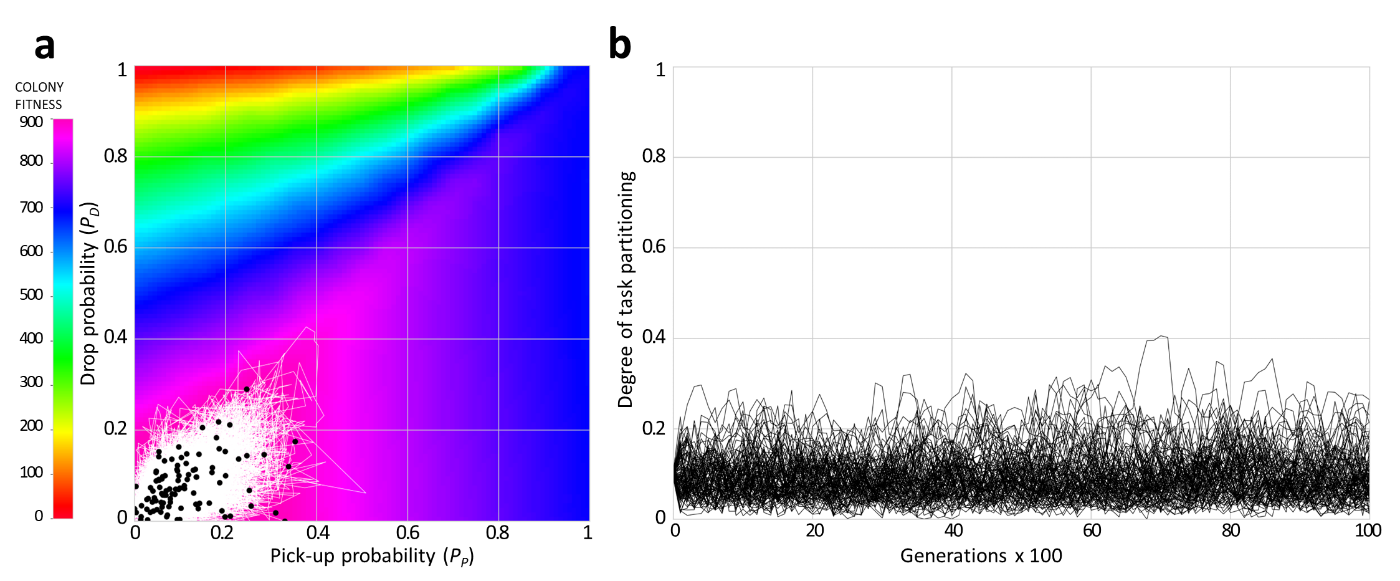


Supplementary Figure S3. **Evolutionary simulation of the agent-based model with tree height = 1.** **a)** Evolutionary trajectories (white lines) of 100 replicate simulations of 50 colonies initialized with *P_P_* and *P_D_* between 0 and 0.2, plotted on the terrestrial fitness landscape. Black dots represent evolved outcomes after 10,000 generations. **b**) Degree of task partitioning, defined as the average between *P_P_* and *P_D_*, is plotted as a function of evolutionary time for the same 100 simulations.


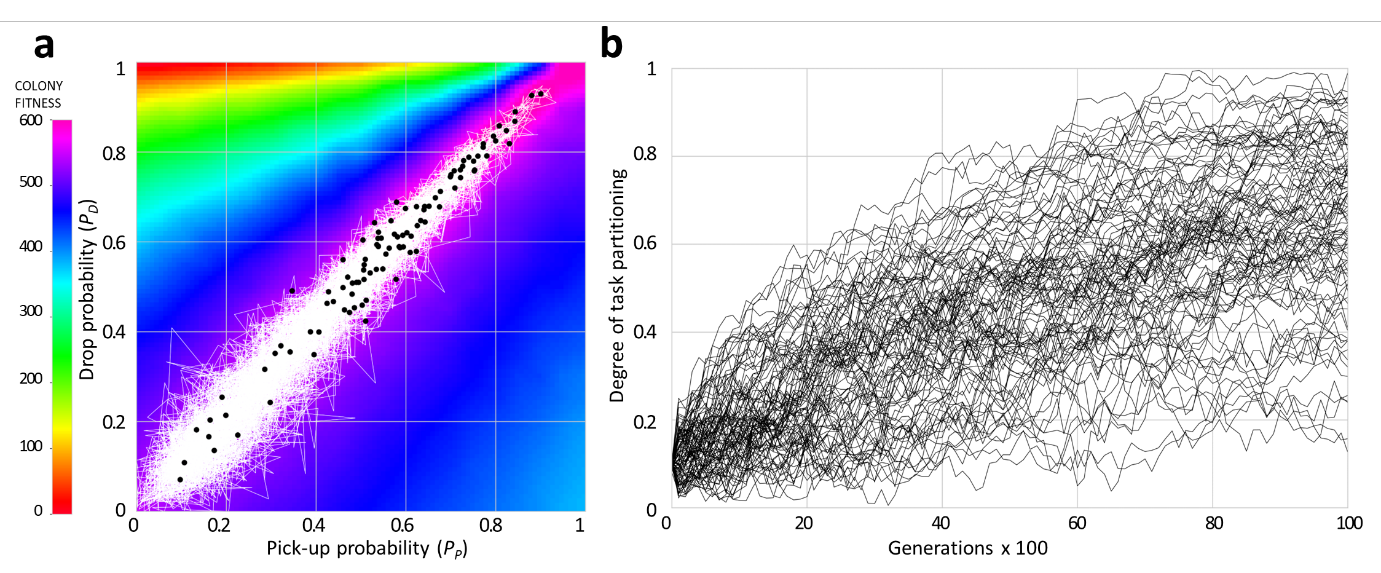


Supplementary Figure S4. **Evolutionary simulation of the agent-based model with tree height = 10. a)** Evolutionary trajectories (white lines) of 100 replicate simulations of 50 colonies initialized with *P_P_* and *P_D_* between 0 and 0.2, plotted on the intermediate fitness landscape. Black dots represent evolved outcomes after 10,000 generations. **b**) Degree of task partitioning, defined as the average between *P_P_* and *P_D_*, is plotted as a function of evolutionary time for the same 100 simulations.


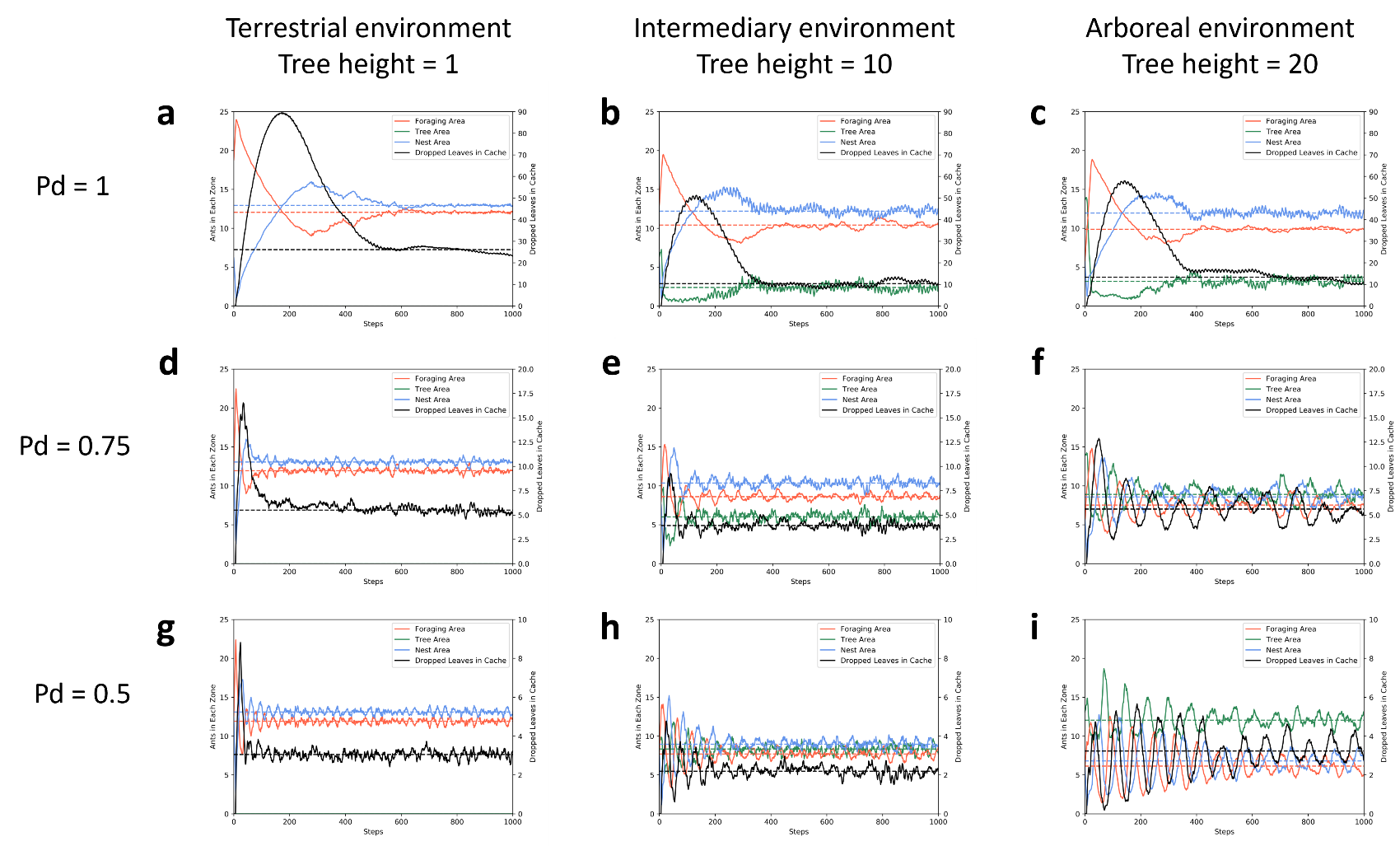


Supplementary Figure S5. **Oscillations due to negative feedback and task switching delay in leaf-cutter ant foraging dynamics.** Foraging ants produce dropped leaves, attracting collector ants which then consume or pick up the dropped leaves. With increasing task switching delay or tree height (moving left to right in the figure), oscillations become more prominent and stable. *P_D_* must be below 1 (below the 1st row) for these oscillations to become apparent. For all figures, *P_P_* = 1 was used. Other simulation variables were consistent with those in Figure 2d-i.


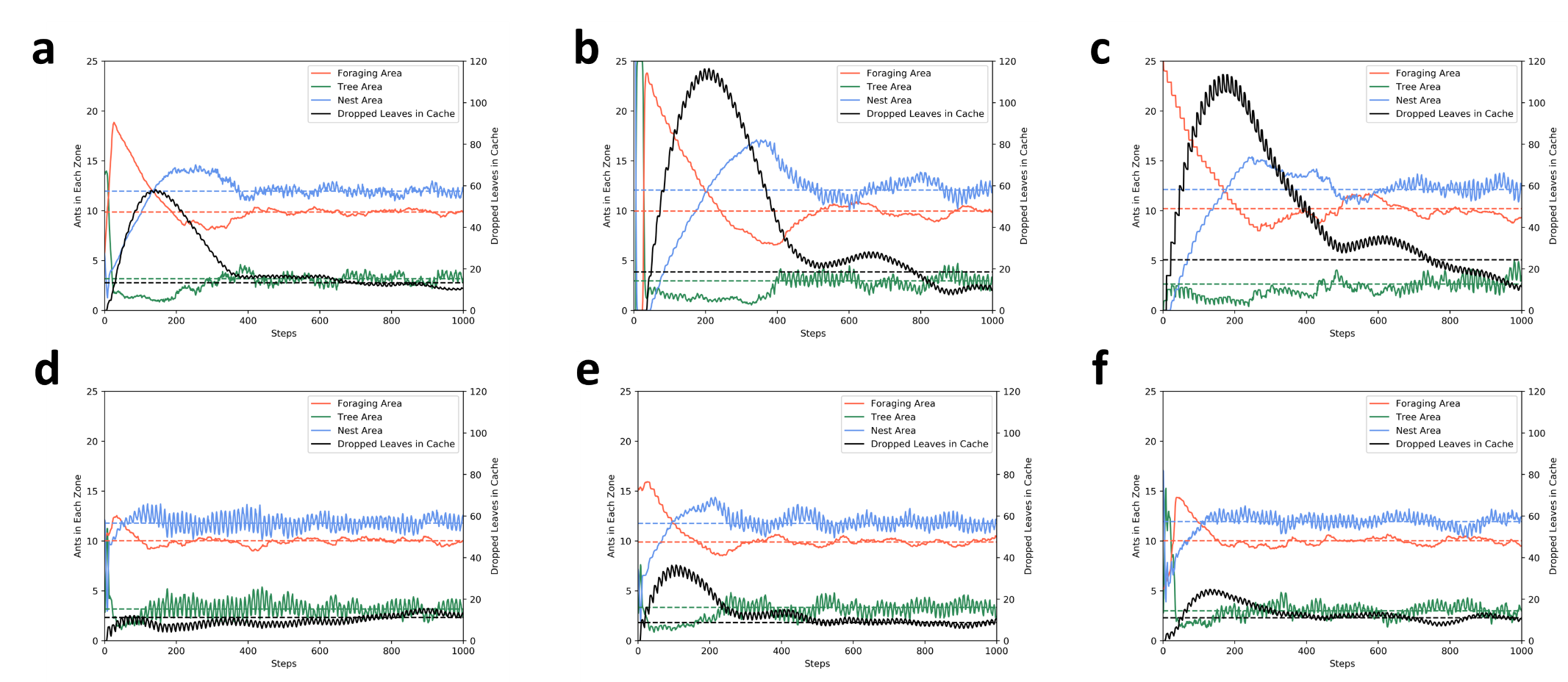


Supplementary Figure S6. **Effect of initialization assumptions on ant movement dynamics**. Variations of how the ants were initialized on the grid for the arboreal environment (tree height = 20) with *P_D_* = *P_P_* = 1. The random initialization scheme (a) is compared with the two extremes where all ants start at the nest location (b) and at the treetop location (c). The ant dynamics at equilibrium (10 foragers, 12 collectors, and 3 ants walking the tree) is used as initialization conditions in (d), slightly varying the forager: collector ratio to 15:7 in (e) and 5:17 in (f).
